# Effects of biochar application and nutrient fluctuation on the growth, and cadmium and nutrient uptake of *Trifolium repens* with different planting densities in Cd-contaminated soils

**DOI:** 10.3389/fpls.2023.1269082

**Published:** 2023-09-20

**Authors:** Wei-Long Zheng, Yan-Fei Wang, Jingya Mo, Pu Zeng, Jiayi Chen, Chenliang Sun

**Affiliations:** ^1^ Zhejiang Provincial Key Laboratory of Plant Evolutionary Ecology and Conservation, School of Life Sciences, Taizhou University, Taizhou, China; ^2^ Taizhou Institute of Product Quality and Safety Inspection, Taizhou, China

**Keywords:** heavy metal, phytoremediation, planting density, pulsed nutrient, white clover

## Abstract

Biochar has been used to remediate contaminated-soil with heavy metals, however, less is known on how biochar interacts with planting density and nutrient fluctuation to affect the remediation. A pot experiment was conducted in the greenhouse to investigate the effects of biochar application (without vs. with 1% biochar, g/g substrate), nutrient fluctuation (constant vs. pulsed) and planting density (1-, 3- and 6-individuals per pot) on the growth, and cadmium (Cd) and nutrient uptake of *Trifolium repens* population. Our results found that the growth of *T. repens* population increased significantly with increasing planting density, and the increment decreased with increasing planting density. Both the Cd and nutrient uptake were higher at higher planting density (e.g., 3- and 6-individuals) than at lower planting density (e.g., 1-individual). Biochar application increased the biomass and shoot Cd uptake, but decreased the ratio of root to shoot and root Cd uptake of *T. repens* population, the effects of which were significantly influenced by planting density. Although nutrient fluctuation had no effect on the growth of *T. repens* population, but its interaction with planting density had significant effects on Cd uptake in tissues. Overall, the effects of biochar application and nutrient fluctuation on the growth and Cd uptake were both influenced by planting density in the present study. Our findings highlight that biochar application and constant nutrient supply at an appropriate planting density, such as planting density of 3-individuals per pot in the present study, could promote the growth, and Cd and nutrient uptake of *T. repens* population.

## Introduction

Soil contamination with heavy metals has become a major concern due to anthropogenic activities such as mining and smelting, chemical industry and agricultural activities (Zhao et al., 2015; Zhong et al., 2020). Cadmium (Cd) is one of the most hazardous heavy metals, which could be easily accumulated in human body through food chain, resulting in the occurrence of many serious diseases such as blood disorders, organ damage and cancer (Liu et al., 2013a; Briffa et al., 2020). Therefore, the environmental-friendly and sustainable remediation technologies are urgently needed, such as the cost-effective and environmental-friendly techniques of biochar and phytoremediation (Yan et al., 2020; Gao et al., 2022; Shen et al., 2022).

Biochar is a carbon-rich material produced by thermochemical decomposition of biomass under anoxic conditions (Cha et al., 2016; Xie et al., 2022). The biochar with its large specific surface area, great porous structure, active functional groups and high cation exchange capacity has been widely used to adsorb heavy metals in soils and water environment (Narayanan et al., 2021; Chen et al., 2022; Gao et al., 2022; Issaka et al., 2022). For instance, many studies indicated that biochar could effectively immobilize Cd in soils by reducing the mobility and bioavailability, through high polarity and/or abundant chemical functional groups, e.g., hydroxyl, carboxyl, phenolic groups, and π electron-rich domain on the aromatic structures (Cai et al., 2020; Tu et al., 2020). These groups give biochar the property of high polarity, thus increasing the interaction between Cd and biochar, resulting in an increase of Cd immobilization (Sumaraj & Padhye, 2017; Wang R. et al., 2018). Additionally, the application of biochar in soils has often been reported to have positive effects on plant growth (e.g., increasing biomass) (Houben et al., 2013; Li et al., 2019). It can be imagined that when the biomass of plants, especially for plants with high biomass and Cd-tolerance, and/or Cd-hyperaccumulators growing in Cd-contaminated soils increase with application of biochar, more Cd would be stored in plants, leading to higher remediation efficiency.

Phytoremediation approach has also been suggested as a promising environmental-friendly technique to remediate soils contaminated with heavy metals (Sarwar et al., 2017; Shen et al., 2022). The main approach is the use of hyperaccumulator plants which could accumulate more (e.g. more than 100 times) metals in shoots compared to normal plants (He et al., 2017). Alternatively, it’s possible to use plants that can produce high biomass and be tolerant to above-threshold soil metal concentrations (Chen et al., 2022a). Generally, the population biomass is regulated by the planting density, which initially proportionally increases, levels off and then remains constant with increasing density (Li et al., 2016). Thus, it is expected that the large and Cd-tolerant plant with appropriate density could produce great biomass, and subsequently immobilize high Cd in tissues. For instance, the high phytoremediation efficiency of heavy metals in *Festuca arundinacea* (Qin et al., 2021), *Eucalyptus globulus* (Luo et al., 2018) and *Typha domingensis* (Viana et al., 2021) with appropriate plant densities.

Due to human-driven global change, most terrestrial ecosystems are experiencing great fluctuations in resource availability (Li et al., 2022; Tao et al., 2023). The fluctuating resource can influence the growth of plants directly by changing the nutrient uptake (Warren et al., 2003), or indirectly by affecting the activities of soil microbes (Carrero-Colón et al., 2006a; Carrero-Colón et al., 2006b), and then subsequently affects the heavy metal uptake of plants. Alternatively, the changed activities of soil microbes induced by pulsed nutrient can directly affect the heavy metal remediation, as microbial bioremediation has been used as an effective technique in the remediation of contaminated-soils with heavy metal (Wang et al., 2020; Xiao et al., 2020a).

*Trifolium repens* L. is an invasive perennial legume with large biomass and strong adaptability. It could grow as a dominant species in heavy metal contaminated soils, which possesses great potential for phytoremediation (Liu et al., 2018; Nandillon et al., 2019). Many previous studies have found great Cd accumulation in *T. repens* (Wang L. et al., 2018; Liu et al., 2019; Xiao et al., 2020c). In this study, we carried out a greenhouse experiment to investigate the interactive effects of planting density, biochar and nutrient fluctuation on the growth, Cd and nutrient of *Trifolium repens* population. We aimed to answer that (1) whether the Cd and nutrient uptake of *Trifolium repens* increased with higher planting density due to the possible increasing biomass; (2) whether the biochar effect was adjusted by nutrient fluctuation, as previous study pointed that the effect of biochar was not significant without nutrient application (Kuppusamy et al., 2016); (3) whether compared with constant nutrient application, pulsed nutrient application can promote more the growth of *Trifolium repens*, and subsequently the greater Cd and nutrient accumulation in tissues.

## Materials and methods

### Seedling preparation

The seed of *T. repens* was collected in the campus of Taizhou University, and then was stored at 4°C until use. On 5 April 2022, the seeds were sown in plastic pots (54 cm long × 28 cm wide × 5 cm deep) filled with peat in a greenhouse at Taizhou University. The temperature and relative humidity in the greenhouse were maintained at 25°C and 80%, respectively.

### Experimental design

**Figure 1:**
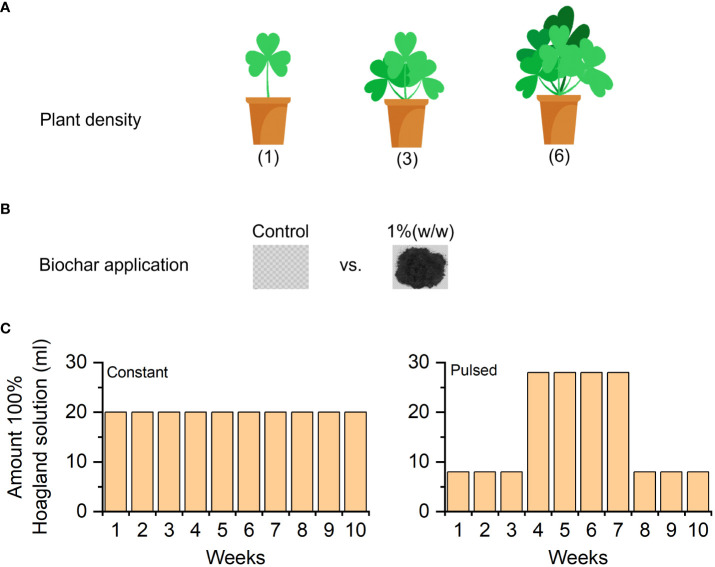
Graphical illustration of the experimental design. The planting density (1-, 3- and 6-individuals) of white clover **(A)**; the application of biochar (without vs. with) **(B)**; and the amount of nutrient solution supplied each week during the 10 weeks of the experiment **(C)**.

A pot experiment was carried out in the same greenhouse used for seed germination and seedling cultivation. The experiment consisted of two levels of biochar application (without vs. with) and two levels of nutrient fluctuation (constant vs. pulsed), crossed with three levels of planting density (1, 3 and 6 individuals). Therefore, there were 2 biochar application × 2 nutrient fluctuation × 3 planting density × 7 replicates = 84 pots in total (**Figure 1**).

The substrate used was a 1:1 (v/v) mixture of local soil and river sand. The local soil was collected in mountainous areas of Taizhou City. It contained 0.39 ± 0.01 (mean ± SE) g kg^-1^ total nitrogen, 0.64 ± 0.04 g kg^-1^ total phosphorus and 10.04 ± 1.03 g kg^-1^ organic carbon, with a CEC of 11.69 ± 1.78 cmol kg^-1^ and a pH of 6.45 ± 0.98. On 5 April 2022, a dose of 1% (w/w, biochar/substrate) biochar was added into 42 pots (19 cm in diameter and 11 cm in height, 2 L) and was mixed with the substrate thoroughly, and the other 42 pots were without biochar application. The biochar was purchased from a market in Zhengzhou, Henan Province, China, which was derived from maize straw and was pyrolyzed at 550°C in a muffle furnace, containing 375.30 ± 67.99 (mean ± SE) g kg^−1^ total carbon and 1.36 ± 0.15 g kg^−1^ total nitrogen, pH 9.76 ± 0.57. On 9 April 2022, to simulate Cd-contaminated substrate, 10 mg kg^-1^ Cd in the form of CdCl_2_ was added in each pot, which was then watered and stirred every day to make sure that Cd was distributed evenly in the substrate. The amount of Cd added is close to the Cd concentration in some contaminated soils in Taizhou, where many electronic waste dismantling industries were located (Wu et al., 2019). On 9 June 2022, for without and with biochar pots, three different densities (i.e., 1, 3 or 6 individuals per pot) of seedlings were randomly transplanted into 14 pots, respectively. During the first two weeks after transplantation, the dead seedlings were replaced immediately. All the pots were watered every two days.

On 4 July 2022 (i.e., four weeks after transplanting), we started to apply the nutrient treatments at weekly intervals for a total of 10 weeks. We applied two nutrient-fluctuation treatments (constant vs. pulsed), using a 100%-strength Hoagland solution (945 mg L^-1^ Ca(NO_3_)_2_·4H_2_O, 607 mg L^-1^ K_2_SO_4_, 115 mg L^-1^ NH_4_H_2_PO_4_, 493 mg L^-1^ MgSO_4_, 20 mg L^-1^ Na_2_-EDTA, 2.86 mg L^-1^ FeSO_4_, 4.5 mg L^-1^H_3_BO_3_, 2.13 mg L^-1^ MnSO_4_, 0.05 mg L^-1^ CuSO_4_, 0.22 mg L^-1^ ZnSO_4_, 0.02 mg L^-1^ (NH_4_)_2_SO_4_). During the experiment, we added a total of 200 ml of the Hoagland solution to pots. For the constant nutrient treatment, we supplied 20 ml of the nutrient solution each week (10 weeks, 200 ml in total). The pulsed treatment consisted of 3 weeks of 8 ml of the nutrient solution per week, followed by 4 weeks of 28 ml of the nutrient solution per week, and again 3 weeks of 8 ml per week (10 weeks, 200 ml in total). To avoid differences in water supply among the two nutrient treatments, we added extra water to the amount of nutrient solution in each treatment to ensure that each pot received a total of 100 ml of water per nutrient application.

### Harvest and measurements

On 11 September 2022 (i.e., 13 weeks after transplanting), we harvested the shoots and roots of all pots. All shoot samples and cleaned root samples were oven-dried at 65°C for 72 h, and then weighed. Then, shoots and roots were separately ground into fine powder and used to measure total Cd concentrations. The total Cd concentrations in shoots and roots were measured by ICP-MS (NexION 2000B, Perkinelmer, USA) after digestion with a mixture of sulfuric and perchloric acid (10:1). We calculated the Cd pool size in shoots, roots and the whole population by multiplying the Cd concentration and the dry biomass together.

### Statistical analysis

Three-way ANOVAs were used to examine the effect of biochar application, nutrient fluctuation, planting density and their interactions on the growth (i.e., shoot biomass, root biomass, total biomass and root: shoot ratio), Cd levels (i.e., Cd concentration in shoots and roots, and Cd pool size in shoots, roots and the whole population), and nutrient levels (i.e., both the concentrations and the pool sizes of N and P in shoots, roots and the whole population), as well as bioavailable Cd, total N and total P in soils. All data were checked for normality using the Kolmogorov-Smirnov test and for homogeneity of variance using Levene’s test. Data for root biomass, root: shoot ratio, Cd concentration in shoots, Cd pool size in roots and the whole population were log (x+1) transformed, and data for N pool size in roots and the whole population were square-root transformed before analysis. All statistical analyses were performed using SPSS software version 22.0 (IBM Corp., Armonk, NY, USA).

## Results

### Growth response

**Figure 2:**
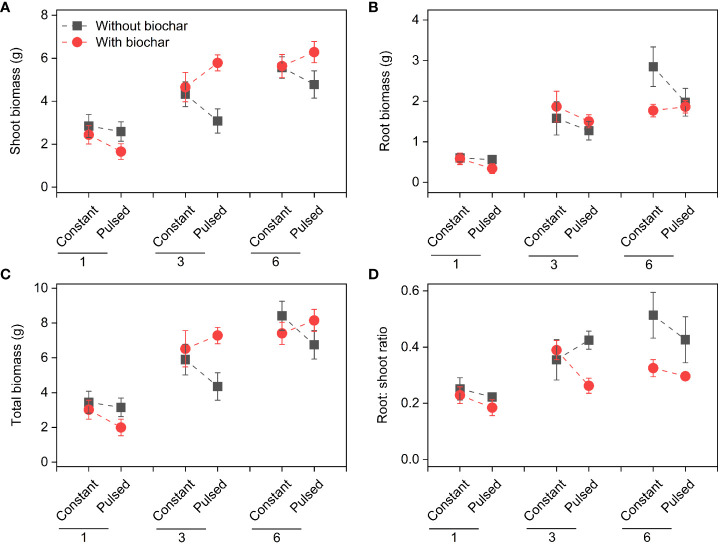
Effects of planting density (1, 3 and 6), biochar application (without vs. with) and nutrient fluctuation (constant vs. pulsed) on shoot biomass **(A)**, root biomass **(B)**, total biomass **(C)** and root: shoot ratio **(D)**.

Shoot biomass, root biomass, total biomass and root: shoot ratio increased significantly with increasing initial planting density (all *P*<0.001; **Figures 2**, **S1**; **Table S1**). Both shoot biomass and total biomass were lower with biochar application than without biochar application at lower planting density (i.e., 1-individual), but the opposite results were observed at higher plant densities (i.e., 3- and 6-individuals) (*P*=0.013 and *P*=0.043 for a B × D interaction, respectively; **Figures 2A, C**, **S1A, C**; **Table S1**). Biochar application significantly decreased the root: shoot ratio (*P*=0.002; **Figures 2D**, **S1D**; **Table S1**). However, nutrient fluctuation had no effect on the growth status of *T. repens* population (**Figures 2**, **S1**; **Table S1**).

### Cd response

**Figure 3:**
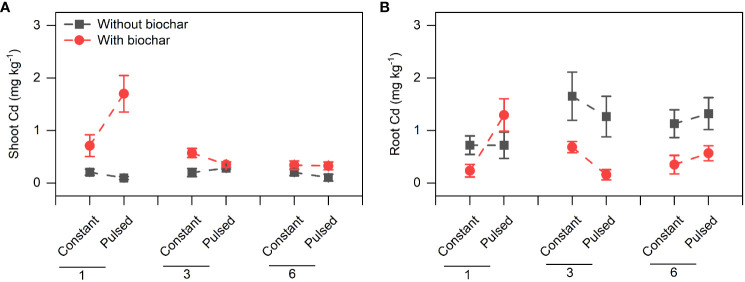
Effects of planting density (1, 3 and 6), biochar application (without vs. with) and nutrient fluctuation (constant vs. pulsed) on Cd concentrations in shoots **(A)** and roots **(B)**.

**Figure 4:**
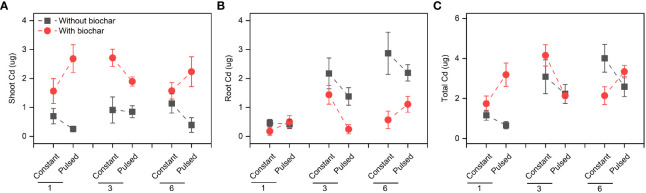
Effects of planting density (1, 3 and 6), biochar application (without vs. with) and nutrient fluctuation (constant vs. pulsed) on Cd pool size in shoots **(A)**, roots **(B)** and the whole population **(C)**.

**Figure 5:**
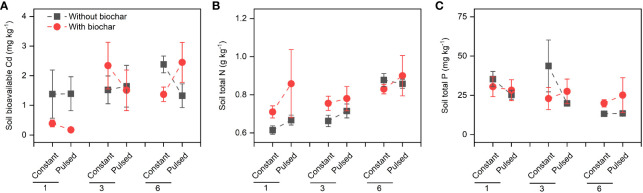
Effects of planting density (1, 3 and 6), biochar application (without vs. with) and nutrient fluctuation (constant vs. pulsed) on soil bioavailable Cd **(A)**, and the concentrations of soil total N **(B)** and soil total P **(C)**.

Biochar application significantly increased the Cd concentration in shoots (+267.80%, *P*<0.001; **Figures 3A**, **S2A**; **Table S2**), but significantly decreased it in roots (-51.77%, *P*<0.001; **Figures 3B**, **S2B**; **Table S2**). However, the magnitude of the effect of biochar application on shoot Cd concentration was higher at lower planting density (+702.41%) than at higher planting density (+101.85%) (*P*<0.001 for a B × D interaction; **Figure 3A**, **S2A**; **Table S2**), and the opposite results were observed for root Cd concentration (*P*=0.013 for a B × D interaction; **Figures 3B**, **S2B**; **Table S2**). Biochar application significantly increased the Cd pool size in shoots (+198.45%, *P*<0.001; **Figures 4A**, **S3A**; **Table S2**), Biochar application had significant effects on the Cd pool size in roots (-57.47%) and the whole population (+21.50%) (all *P*<0.001; **Figures 4B, C**, **S3B, C**; **Table S2**), and the biochar effects were significantly influenced by planting density (*P*=0.014 and *P*=0.001 for a B × D interaction, respectively; **Figures 4B, C**, **S3B, C**; **Table S2**). Both Cd pool size in roots and the whole population were significantly lower at lower planting density than at higher planting density (all *P*<0.001; **Figures 4B, C**, **S3B, C**; **Table S2**). There were significantly interactive effects between nutrient fluctuation and planting density on Cd concentration in shoots (*P*=0.041; **Figures 3A** **S4A**; **Table S2**) and roots (*P*=0.029; **Figures 3B**, **S4B**; **Table S2**), and the Cd pool size in roots (*P*=0.007; **Figures 4B**, **S5B**; **Table S2**). Bioavailable Cd in soils was not affected by either biochar application or nutrient fluctuation, however, it was significantly lower at lower planting density (i.e., 1-individual) than at higher planting density (i.e., 1- and 3-individuals) (*P*=0.018; **Figure 5A**; **Table S3**).

### Nutrient response

**Figure 6:**
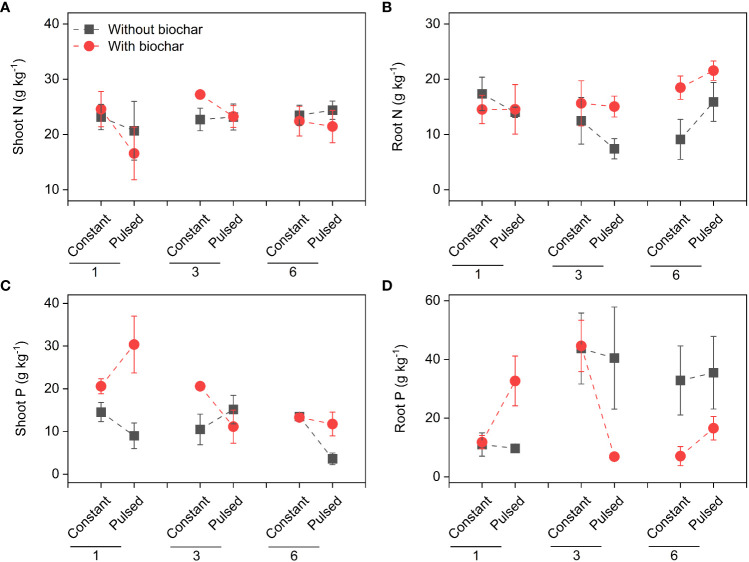
Effects of planting density (1, 3 and 6), biochar application (without vs. with) and nutrient fluctuation (constant vs. pulsed) on N concentrations in shoots **(A)** and roots **(B)**, and P concentrations in shoots **(C)** and roots **(D)**.

**Figure 7:**
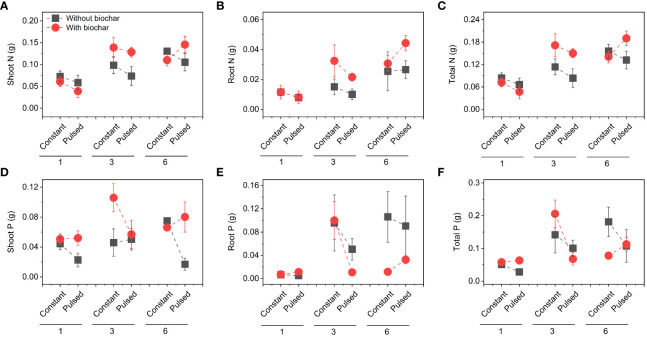
Effects of planting density (1, 3 and 6), biochar application (without vs. with) and nutrient fluctuation (constant vs. pulsed) on N pool size in shoots **(A)**, roots **(B)** and the whole population **(C)**, and P pool size in shoots **(D)**, roots **(E)** and the whole population **(F)**.

Neither the concentrations of total N nor total P in soils were affected by either biochar application or nutrient fluctuation (all *P* > 0.05; **Figures 5B, C**; **Table S3**). However, the concentration of soil total N was significantly higher at higher planting density (i.e., 6-individuals) than at lower planting density (i.e., 1-individual) (*P*=0.003; **Figure 5B**; **Table S3**), and the concentration of soi total P was the opposite (*P*=0.015; **Figure 5C**; **Table S3**). The concentration of tissue N was not affected by planting density, biochar or nutrient fluctuation (all *P* > 0.05; **Figures 6A, B**; **Table S4**), except the increased concentration of root N with biochar application (*P*=0.03; **Figure 6B**; **Table S4**). The concentration of P in shoots was lower at higher planting density (i.e., 3- and 6-individuals) than at lower planting density (i.e., 1-individual), and the concentration of P in roots was the opposite (*P*=0.002 and *P*=0.025; **Figures 6C, D**; **Table S4**). Both the pool sizes of N and P in shoots, roots and the whole population were higher at higher planting density (i.e., 3- and 6-individuals) than at lower planting density (i.e., 1-individual) (all *P* < 0.05; **Figures 7A–F**; **Table S5**).

## Discussion

We found that the biomass increased significantly with increasing planting density (**Figures 2**, **S1**), which is consistent with many previous studies (Hagiwara et al., 2010; Li et al., 2013; Springer, 2021; Sun et al., 2022), because that higher planting density can produce higher productivity with sufficient light, water and nutrient (Chu et al., 2008). However, the growth efficiency of *T. repens* population was higher at the planting density of 3-individuals (e.g., + 107.18% for total biomass from 1-individual to 3-individuals) than of 6-individuals (e.g., + 27.70% for total biomass from 3-individuals to 6-individuals). This could be largely due to the application of biochar (significant interaction effect between planting density and biochar application; **Figures 2**, **S1**; **Table S1**). The growth efficiency increased with increasing planting density without biochar application, whereas it was higher at planting density of 3-individuals than of 6-individuals with biochar application (**Figure S1**). Additionally, when the planting density was 3-individuals, biochar application significantly increased the shoot biomass and total biomass, but this increase disappeared when the planting density was 6-individuals (**Figures 2**, **S1**). Additionally, we found that biochar application significantly decreased the ratio of root to shoot, especially when the planting density was 6-individuals (**Figures 2**, **S1**). These results indicate that seriously competition for resources between individuals at planting density of 6-individuals with biochar application may occur. Previous studies have suggested that biochar application could promote the plant growth, due to its benefits such as increased nutrient supply, water conservation and microbial activity (Sohi et al., 2010; Liu et al., 2013b). However, our results indicate that the effect of biochar application on the growth of plant population could be adjusted by planting density, and the planting density of 3-individuals could be an appropriate density for the plant growth. During the vegetative stage of plants, the levels of N and P in tissues represent the levels of nitrogen compounds and phosphorous compounds, especially protein and nucleic acid, respectively, and their levels could reflect the growth status to some extent (Chapin, 1980). In this study, under the treatment of biochar application, the higher growth efficiency of *T. repens* population at the planting density of 3-individuals than that at the planting density of 6-individuals might be due to the higher N concentrations and pool sizes in shoots and roots (**Figures 6A, B**, **7A, B**). Surprisingly, neither nutrient fluctuation alone nor its interaction with density or biochar did affect the growth of *T. repens* population (**Figure 2**; **Table S1**). This indicates that *T. repens* might not be sensitive to the nutrient fluctuation on the one hand. On the other hand, the nutrient dose (100%-strength Hoagland, 200 ml pot^-1^) in the present study might be sufficient for the need of the growth of *T. repens* population, and then the fluctuating supply was ineffective, which can be proved by no significant differences of N and P concentration in soils between the application of constant nutrient and pulsed nutrient (**Figures 5B, C**; **Table S3**).

We found that the concentration of Cd in shoots was higher with biochar application than without biochar application, however, the difference decreased with increasing planting density (**Figure S2A**; **Table S2**). Under biochar application, the decrease of Cd concentration in shoots with increasing planting density might be due to the dilution effect with large biomass (**Figure 2A**). However, this explanation was not applicable to the unchanged Cd concentration without biochar application. Biochar application increased the Cd uptake in shoots but decreased it in roots, however, the effect of biochar application on Cd uptake was also adjusted by planting density. Biochar application benefited more the Cd uptake of *T. repens* population with lower planting density (i.e., 1-individual) in comparison to higher planting density (i.e., 3- and 6-individuals) (**Figures S2**, **S3**). This result indicates that increasing planting density might decrease the Cd uptake efficiency, although the biomass significantly increased. Overall, our results indicate that biochar application can largely promote the Cd uptake of *T. repens* population, which have been found in many previous studies (Wang et al., 2019; Cai et al., 2020; Tu et al., 2020; Xiao et al., 2020b), however, this promotion in the present study depends on the planting density. Generally, rhizobium could fix and provide large amount of the nitrogen nutrients to *T. repens*, and poor nitrogen nutrients in soil could promote the nitrogen fixation (Hoglund and Brock, 1987). In this study, at the early stage, rhizobium might not provide enough N at the planting density of 1-individual due to the unstable soil microbial composition in comparison to the planting density of 3- and 6-individuals. Meanwhile, biochar could not only immobilize Cd, but also absorb N, which could lead to the shortage of N in soils (Kuppusamy et al., 2016). When the soils were supplied with pulsed nutrients (especially low volumes of Hoagland solution for 3 weeks at the early stage), *T. repens* has to response to the N shortage and improve the uptake efficiency of N from the soils, which inevitably absorb more Cd into the plants. Alternatively, higher planting density is likely to cause strong intraspecific competition for light, nutrients and water in a certain spatial range, and in this circumstance, biochar application might have neutral and even negative impact. Biochar application significantly decreased Cd uptake in roots, and the reduction was greater at the planting density of 6-individuals than 3- and 1-individual(s), which possibly resulted from the lower ratio of root to shoot. Obviously, fewer root systems absorbed less Cd. Surprisingly, the soil bioavailable Cd increased under the circumstance that more Cd was accumulated in the *T. repens* population at higher planting density (i.e., 3- and 6-individuals). We guess that compared with 1-individual, more individuals in pots could change the soil properties more, which may lead to some chemical reactions or microbial activities that resulting in more bioavailable Cd.

Although nutrient fluctuation had no effect on the growth of *T. repens* population, but its interaction with planting density had significant effects on Cd uptake in tissues (**Figure S5**; **Table S2**). Constant nutrient promoted Cd uptake in roots and the whole population at planting density of 3-individuals, however, this promotion decreased from planting density of 3-individuals to 6-individuals, which might mainly due to higher root Cd pool size (**Figures 4B**, **S5B**). We calculated average individual plant weight for each planting density treatment (the average biomass divided by the number of individual(s)) in constant and pulsed nutrients, respectively. The average root weight in the treatment of constant nutrients (0.66 g) at the planting density was higher than that of pulsed nutrients (0.47 g), thus more Cd was absorbed. Pulsed nutrient increased the Cd uptake in roots and the whole population with increasing planting density from 1-individual to 6-individuals (**Figures S5B, C**). But there was no significant difference for Cd uptake at the planting density of 6-indivduals between constant nutrient and pulsed nutrient (**Figure S5**). Thus, our results suggest that constant nutrient could promote the Cd uptake of *T. repens* population at an appropriate planting density, such as planting density of 3-individuals in the present study. The nutrient (N and P) uptake of *T. repens* population was higher at higher planting density (i.e., 3- and 6-individuals) than at lower planting density (i.e., 1-individual), which could be due to the higher biomass (**Figure 2**). At planting density of 3- and 6-individuals, biochar application significantly increased root N uptake (**Figures 5B**, **6B**) but decreased root P uptake (**Figures 6D**, **7E**), which indicates that biochar promotes more N uptake in comparison to P uptake.

We conclude that the growth and Cd and nutrient uptake of *T. repens* population increased with increasing planting density. The effects of biochar and nutrient fluctuation on the growth and Cd uptake of *T. repens* population were influenced by planting density. With biochar application, the shoot biomass and Cd uptake were increased more at the planting density of 3-individuals. Constant nutrient had no effect on biomass, but significantly increased root Cd uptake of *T. repens* at the planting density of 3-individuals. Thus, our results suggest that an appropriate planting density, such as the planting density of 3-individuals per pot in the present study, together with biochar and constant nutrients could promote the growth and Cd uptake of *T. repens* population.

## Data availability statement

The raw data supporting the conclusions of this article will be made available by the authors, without undue reservation.

## Author contributions

W-LZ: Conceptualization, Funding acquisition, Writing – original draft. Y-FW: Methodology, Resources, Writing – review & editing. JM: Data curation, Methodology, Writing – review & editing. PZ: Data curation, Methodology, Writing – review & editing. JC: Data curation, Methodology, Writing – review & editing. CS: Data curation, Methodology, Writing – review & editing.

## Supplementary material

Supplementary Table 1 Three-way ANOVA effects of planting density (1, 3 and 6), biochar application (without vs. with) and nutrient fluctuation (constant vs. pulsed) on shoot biomass, root biomass, total biomass and root: shoot ratio.

Supplementary Table 2 Three-way ANOVA effects of planting density (1, 3 and 6), biochar application (without vs. with) and nutrient fluctuation (constant vs. pulsed) on DTPA-Cd in soils, Cd concentrations in shoots and roots, Cd pool size in shoots, roots and the whole population.

Supplementary Table 3 Three-way ANOVA effects of planting density (1, 3 and 6), biochar application (without vs. with) and nutrient fluctuation (constant vs. pulsed) on soil bioavailable Cd, and the concentrations of soil total N and soil total P.

Supplementary Table 4 Three-way ANOVA effects of planting density (1, 3 and 6), biochar application (without vs. with) and nutrient fluctuation (constant vs. pulsed) on the concentrations of N and P in shoots and roots.

Supplementary Table 5 Three-way ANOVA effects of planting density (1, 3 and 6), biochar application (without vs. with) and nutrient fluctuation (constant vs. pulsed) on the pool sizes of N and P in shoots, roots and the whole population.

Supplementary Figure 1 Effects of planting density (1, 3 and 6) and biochar application (without vs. with), on shoot biomass **(A)**, root biomass **(B)**, total biomass **(C)** and root: shoot ratio **(D)**.

Supplementary Figure 2 Effects of planting density (1, 3 and 6) and biochar application (without vs. with) on Cd concentrations in shoots **(A)** and roots **(B)**.

Supplementary Figure 3 Effects of planting density (1, 3 and 6) and biochar application (without vs. with) on Cd pool size in shoots **(A)**, roots **(B)** and the whole population **(C)**.

Supplementary Figure 4 Effects of planting density (1, 3 and 6) and nutrient fluctuation (constant vs. pulsed) on Cd concentrations in shoots **(A)** and roots **(B)**.

Supplementary Figure 5 Effects of planting density (1, 3 and 6) and nutrient fluctuation (constant vs. pulsed) on Cd pool size in shoots **(A)**, roots **(B)** and the whole population **(C)**.
