# Supplementary material for: Effects of biochar application and nutrient fluctuation on the growth, and cadmium and nutrient uptake of Trifolium repens with different planting densities in Cd-contaminated soils
Source: Front Plant Sci. 2023 Sep 20;14:1269082. doi: 10.3389/fpls.2023.1269082 (PMC10548119; doi:10.3389/fpls.2023.1269082)
Supplement: Supplementary Table 1 — Three-way ANOVA effects of planting density (1, 3 and 6), biochar application (without vs. with) and nutrient fluctuation (constant vs. pulsed) on shoot biomass, root biomass, total biomass and root: shoot ratio. [file DataSheet_1.pdf]

Table S1 Three-way ANOVA effects of planting density (1, 3 and 6), biochar application (without vs. with) and nutrient fluctuation (constant vs. pulsed) on shoot biomass, root biomass, total biomass and root: shoot ratio.

		Shoot biomass		Root biomass		Total biomass		Root: shoot ratio	
	d.f.	*F*	*P*	*F*	*P*	*F*	*P*	*F*	*P*
Density (D)	2	38.66	**<0.001**	57.35	**<0.001**	46.32	**<0.001**	17.65	**<0.001**
Biochar (B)	1	3.27	0.075	0.58	0.449	0.90	0.345	10.33	**0.002**
Nutrient (N)	1	0.51	0.476	3.30	0.073	1.50	0.224	2.58	0.112
D × B	2	4.58	**0.013**	2.22	0.116	3.30	**0.043**	1.81	0.171
D × N	2	0.25	0.776	0.01	0.990	0.04	0.963	0.11	0.895
B × N	1	3.33	0.072	0.18	0.676	2.62	0.110	1.36	0.247
D × B × N	2	2.02	0.140	1.34	0.267	1.56	0.217	2.42	0.097

Table S2 Three-way ANOVA effects of planting density (1, 3 and 6), biochar application (without vs. with) and nutrient fluctuation (constant vs. pulsed) on DTPA-Cd in soils, Cd concentrations in shoots and roots, Cd pool size in shoots, roots and the whole population.

		Shoot Cd concentration		Root Cd concentration		Shoot Cd pool size		Root Cd pool size		Total Cd pool size	
	d.f.	*F*	*P*	*F*	*P*	*F*	*P*	*F*	*P*	*F*	*P*
Density (D)	2	11.01	**<0.001**	0.58	0.561	0.89	0.416	19.66	**<0.001**	15.01	**<0.001**
Biochar (B)	1	57.04	**<0.001**	15.58	**<0.001**	51.43	**<0.001**	26.20	**<0.001**	7.15	**0.010**
Nutrient (N)	1	0.62	0.435	0.38	0.541	0.06	0.809	1.36	0.249	1.17	0.285
D × B	2	13.76	**<0.001**	4.79	**0.013**	0.57	0.571	4.69	**0.014**	7.64	**0.001**
D × N	2	3.42	**0.041**	3.81	**0.029**	1.31	0.280	5.60	**0.007**	3.07	0.056
B × N	1	2.78	0.102	1.12	0.295	3.56	0.065	0.44	0.508	5.91	**0.019**
D × B × N	2	7.85	**0.001**	1.59	0.214	3.66	**0.033**	2.77	0.073	4.84	**0.012**

Table S3 Three-way ANOVA effects of planting density (1, 3 and 6), biochar application (without vs. with) and nutrient fluctuation (constant vs. pulsed) on soil bioavailable Cd, and the concentrations of soil total N and soil total P.

		Soil bioavailable Cd		Soil total N		Soil total P	
	d.f.	*F*	*P*	*F*	*P*	*F*	*P*
Density (D)	2	4.36	**0.018**	7.03	**0.003**	4.77	**0.015**
Biochar (B)	1	0.46	0.501	3.27	0.079	0.08	0.782
Nutrient (N)	1	0.97	0.329	1.46	0.235	1.11	0.298
D × B	2	1.15	0.324	1.08	0.351	1.72	0.194
D × N	2	0.06	0.942	0.28	0.755	0.37	0.690
B × N	1	<0.01	0.949	0.09	0.760	1.28	0.265
D × B × N	2	2.03	0.142	0.22	0.803	0.59	0.560

Table S4 Three-way ANOVA effects of planting density (1, 3 and 6), biochar application (without vs. with) and nutrient fluctuation (constant vs. pulsed) on the concentrations of N and P in shoots and roots.

		Shoot N		Root N		Shoot P		Root P	
	d.f.	*F*	*P*	*F*	*P*	*F*	*P*	*F*	*P*
Density (D)	2	0.97	0.388	1.47	0.240	7.01	**0.002**	3.99	**0.025**
Biochar (B)	1	0.04	0.836	4.99	**0.030**	15.33	**<0.001**	3.81	0.057
Nutrient (N)	1	1.95	0.169	0.01	0.932	1.29	0.262	0.06	0.801
D × B	2	0.64	0.534	2.20	0.122	3.73	**0.031**	7.68	**0.001**
D × N	2	0.83	0.441	1.88	0.163	1.66	0.202	7.92	**0.001**
B × N	1	1.37	0.247	0.15	0.696	0.81	0.372	0.02	0.883
D × B × N	2	0.10	0.901	0.53	0.591	6.34	**0.004**	4.06	**0.024**

Table S5 Three-way ANOVA effects of planting density (1, 3 and 6), biochar application (without vs. with) and nutrient fluctuation (constant vs. pulsed) on the pool sizes of N and P in shoots, roots and the whole population.

		Shoot N		Root N		Total N		Shoot P		Root P		Total P	
	d.f.	*F*	*P*	*F*	*P*	*F*	*P*	*F*	*P*	*F*	*P*	*F*	*P*
Density (D)	2	17.72	**<0.001**	15.73	**<0.001**	20.65	**<0.001**	3.20	**0.050**	6.33	**0.004**	9.62	**<0.001**
Biochar (B)	1	2.18	0.146	6.29	**0.016**	3.94	0.053	11.96	**0.001**	4.06	**0.049**	0.10	0.759
Nutrient (N)	1	1.14	0.291	0.29	0.592	1.01	0.319	5.83	**0.020**	2.03	0.160	5.89	**0.019**
D × B	2	3.77	**0.030**	2.74	0.075	3.86	**0.028**	0.36	0.700	2.55	0.088	1.54	0.226
D × N	2	0.66	0.522	1.93	0.156	1.13	0.333	0.28	0.756	2.45	0.097	1.42	0.252
B × N	1	1.41	0.241	0.03	0.861	1.18	0.282	0.87	0.357	0.00	0.974	0.32	0.575
D × B × N	2	1.14	0.329	0.16	0.856	1.17	0.319	5.85	**0.005**	0.74	0.480	3.69	**0.032**

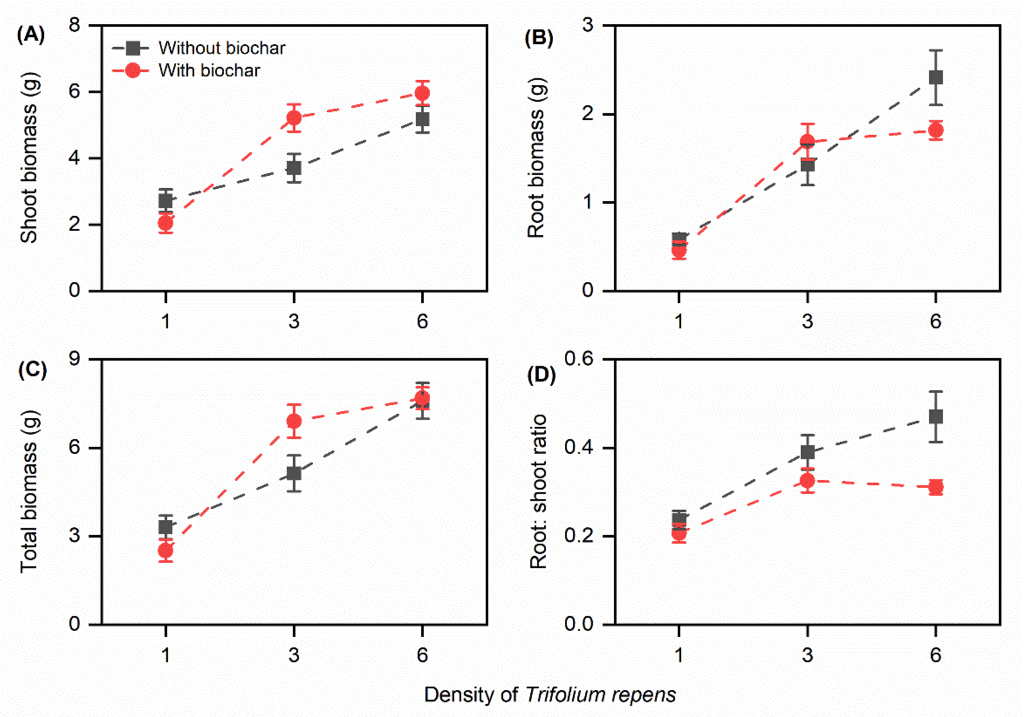

Figure S1 Effects of planting density (1, 3 and 6) and biochar application (without vs. with), on shoot biomass (A), root biomass (B), total biomass (C) and root: shoot ratio (D).

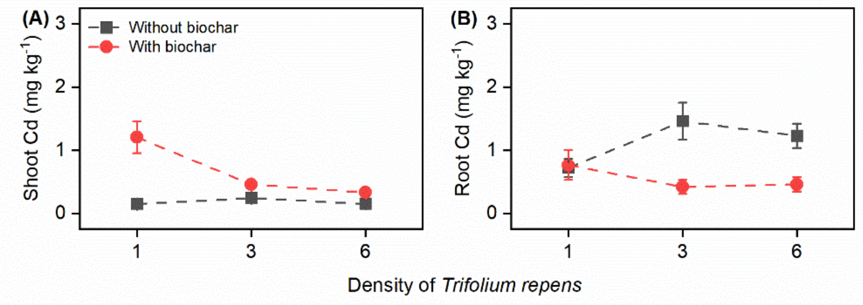

Figure S2 Effects of planting density (1, 3 and 6) and biochar application (without vs. with) on Cd concentrations in shoots (A) and roots (B).

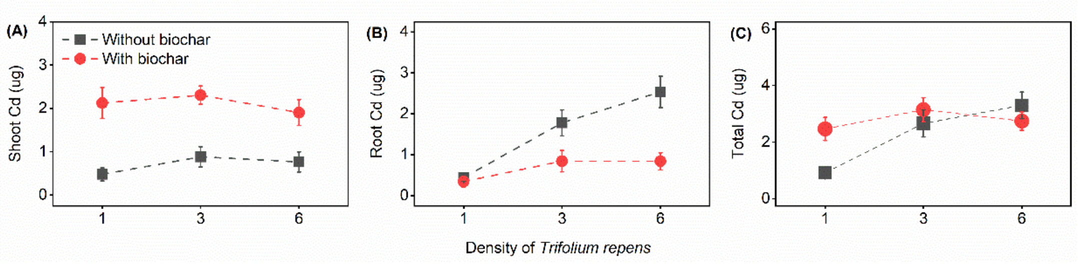

Figure S3 Effects of planting density (1, 3 and 6) and biochar application (without vs. with) on Cd pool size in shoots (A), roots (B) and the whole population (C).

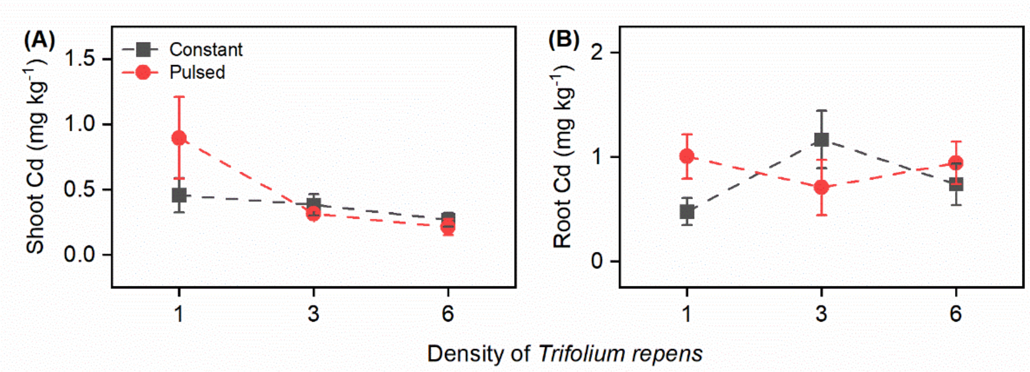

Figure S4 Effects of planting density (1, 3 and 6) and nutrient fluctuation (constant vs. pulsed) on Cd concentrations in shoots (A) and roots (B).

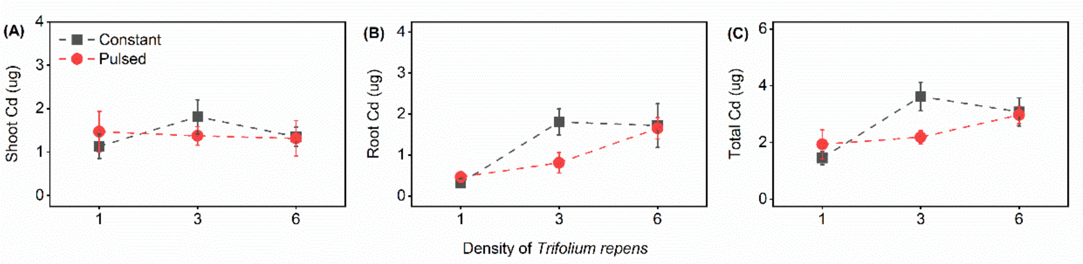

Figure S5 Effects of planting density (1, 3 and 6) and nutrient fluctuation (constant vs. pulsed) on Cd pool size in shoots (A), roots (B) and the whole population (C).
